# A functional genetic screen identifies the Mediator complex as essential for SSX2-induced senescence

**DOI:** 10.1038/s41419-019-2068-1

**Published:** 2019-11-06

**Authors:** Nadine H. Brückmann, Sofie N. Bennedsen, Pascal H. G. Duijf, Mikkel G. Terp, Mads Thomassen, Martin Larsen, Christina B. Pedersen, Torben Kruse, Nicolas Alcaraz, Henrik J. Ditzel, Morten F. Gjerstorff

**Affiliations:** 1 Department of Cancer and Inflammation Research, Institute for Molecular Medicine, University of Southern Denmark, Odense, Denmark; 2 Institute of Health and Biomedical Innovation, Faculty of Health, School of Biomedical Sciences, Queensland University of Technology, Brisbane, QLD Australia; 3 University of Queensland Diamantina Institute, The University of Queensland, Translational Research Institute, 37 Kent Street, Brisbane, QLD 4102 Australia; 4 Department of Clinical Genetics, Odense University Hospital, Odense, Denmark; 5 Department of Oncology, Odense University Hospital, Odense, Denmark; 6 Academy of Geriatric Cancer Research (AgeCare), Odense University Hospital, Odense, Denmark

**Keywords:** Oncogenes, Senescence

## Abstract

The senescence response to oncogenes is believed to be a barrier to oncogenic transformation in premalignant lesions, and describing the mechanisms by which tumor cells evade this response is important for early diagnosis and treatment. The male germ cell-associated protein SSX2 is ectopically expressed in many types of cancer and is functionally involved in regulating chromatin structure and supporting cell proliferation. Similar to many well-characterized oncogenes, SSX2 has the ability to induce senescence in cells. In this study, we performed a functional genetic screen to identify proteins implicated in SSX2-induced senescence and identified several subunits of the Mediator complex, which is central in regulating RNA polymerase-mediated transcription. Further experiments showed that reduced levels of MED1, MED4, and MED14 perturbed the development of senescence in *SSX2*-expressing cells. In contrast, knockdown of MED1 did not prevent development of B-Raf- and Epirubicin-induced senescence, suggesting that Mediator may be specifically linked to the cellular functions of SSX2 that may lead to development of senescence or be central in a SSX2-specific senescence response. Indeed, immunostaining of melanoma tumors, which often express SSX proteins, exhibited altered levels of MED1 compared to benign nevi. Similarly, RNA-seq analysis suggested that MED1, MED4, and MED14 were downregulated in some tumors, while upregulated in others. In conclusion, our study reveals the Mediator complex as essential for SSX2-induced senescence and suggests that changes in Mediator activity could be instrumental for tumorigenesis.

## Introduction

Cellular senescence is an aging-related growth arrest of cells that contributes to the pathology of many diseases of the elderly, such as diabetes, cardiovascular disease, and cancer^1,2^. Senescence was first described as a response to cell division-related telomere erosion in human diploid cells (i.e., replicative senescence), but over the years different types of cellular stress have been demonstrated to induce senescence, including DNA damage, oxidative stress, oncogene activation, and certain chemotherapeutic drugs^1,2^. Although much has been learned about the initiating factors of senescence and the phenotype of senescent cells, the molecular basis of the senescence response is still largely uncharacterized. Since the economic and social burdens associated with demographic shifts in many countries have prompted the need for better diagnostics and treatment of age-related diseases, we need a better understanding of the senescence response.

In cancer, senescence may act as a powerful barrier to cellular transformation by blocking the effects of activated oncogenes. This is supported by in vivo observations of nevi, which invariably express activated oncogenes together with classical senescence-associated markers (e.g., senescence-associated beta-galactosidase activity and p16 activation)^3–7^. Furthermore, nevi show no signs of telomere attrition, suggesting that the senescence response is driven by oncogene expression rather than loss of replicative potential^7^. Oncogene-induced senescence has been studied in several in vitro models with strong oncogenes such as Ras, B-Raf, Cyclin E, and Myc, which suggest that replication stress may be instrumental for development of this phenotype^8,9^.

We recently demonstrated that SSX2 induces senescence in different types of cells, as determined by classical senescence features, including enlargement of the cytoplasm, cell growth arrest, enhanced B-galactosidase activity, and DNA double strand breaks^10^. SSX proteins are germ cell-specific proteins that are expressed at the spermatogonial stage of spermatogenesis^11^. They are also ectopically expressed in many types of tumors, such as 40% of melanomas and up to 65% of breast cancers^12,13^. The SSX family comprise nine highly identical members, which are most likely redundant in their cellular functions^14^. SSX proteins belong to the cancer/testis (CT) antigen group of tumor antigens, and T-cell and antibody responses have been detected in cancer patients^15^. The role of SSX proteins in tumor formation and progression remains largely elusive. We have demonstrated that knockdown of SSX genes in melanoma cancer cells significantly reduces cellular proliferation^10^ and SSX proteins were shown to activate several important mitogenic pathways, such as MAPK and Wnt^16^. The role of SSX proteins in supporting cell proliferation and their ability to induce senescence suggest that these CT antigens may have oncogenic potential. SSX proteins are DNA-binding factors that regulate chromatin structure and Polycomb function^14,15,17,18^. They contain an SSX repression (SSXRD) domain and a Kruppel-associated box (KRAB), which seem to be important for chromatin-binding and -rearrangement, respectively^14^. However, the specific mechanism by which SSX proteins regulate chromatin structure remains unknown.

In this study, we performed a functional genetic screen to identify factors essential for SSX2-induced senescence in order to further understand the molecular functions of SSX2 and the cellular response to SSX2. The results showed that several subunits of the Mediator complex are important for SSX2-induced senescence. The Mediator complex is directly implicated in RNA polymerase II-mediated transcription, and its main function is to transduce signals from the transcription activators bound to enhancer regions to the transcription machinery^19^. Thus, Mediator is important for integration of signals from different signaling pathways that impinge on chromatin to regulate gene transcription. In addition, the Mediator complex seems to play a role in chromatin structure and the spatial positioning of chromatin in the nucleus. It has been demonstrated that Mediator interacts with the SWI/SNF and CHD1 chromatin remodeling complexes^20–22^ and is important for heterochromatin formation^23^. Mediator is also important for the formation and stabilization of chromatin loops^24–26^ and for regulation of gene expression by association with the nuclear pore complex^27^. The human Mediator complex generally consists of 26 subunits, but the complex seems highly flexible in structure^28^. Several studies have indicated that subsets of endogenous Mediator complexes may lack specific subunits and therefore acquire different functions. For instance, the thyroid hormone receptor binds the complex through MED1, and *MED1* knockout cells have impaired activation of hormone receptor target genes^29^, but Mediator complexes lacking MED1 could integrate signals from transcription factors that bind other subunits. In addition, the Mediator complex associates with additional factors, such as the four subunit CDK8 module, which dramatically alters its structure and function^28^. These results suggest that the transcriptional integration of different signaling events and associated transcription factors rely on specific components and possible compositions of the Mediator complex. In accordance, Mediator complexes with a simplified subunit composition have been identified in differentiated cells^30^. Mediator subunits have been shown to exhibit cancer-specific transcription profiles that indicate disease-related shifts in Mediator complex composition^31^. Furthermore, different subunits have been implicated in cancer development or progression^32^.

As described above, the Mediator complex is important in multiple cellular functions and there is growing evidence for a role in cancer. In this study, we describe for the first time a role for Mediator in SSX2-mediated senescence.

## Results

### A functional genetic screen identified potential mediators of SSX2-induced senescence

**Fig. 1:**
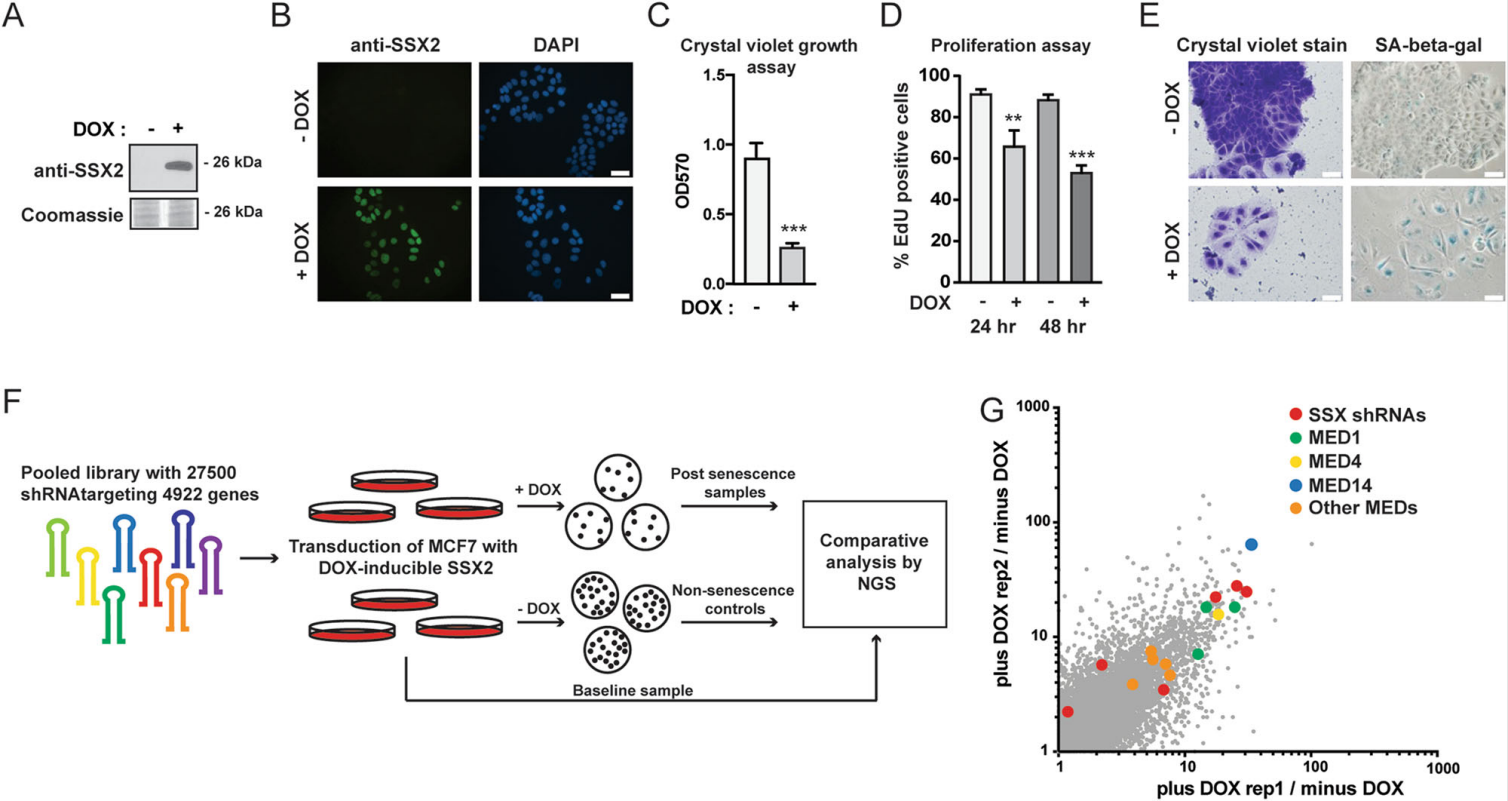
Functional genetic screening for identification of genes essential for SSX2-induced senescence. **a**–**e** MCF7 cells undergo senescence in response to doxycycline-inducible expression of SSX2. This cell model has been established previously^10^. Expression of SSX2 was validated by Western blotting (**a**) and immunocytochemical staining (**b**). For Western blot, Coomassie staining of the membrane was included as loading control. After 7 days of culture with doxycycline, the cells exhibited loss of proliferation as determined by a crystal violet staining assay. Stained cells were solubilized and staining levels were quantified at OD570 (**c**). Proliferation assays were done with EdU incorporation for 24 h (**d**). The cells also acquired a morphology typical for senescent cells (i.e., enlarged cell size and cytoplasm) and exhibited beta-galactosidase activity (**e**). **f** MCF7 cells with doxycycline-regulated SSX2 were transduced with a pooled shRNA library containing 27,500 shRNA targeting 4922 genes and cultures with or without doxycycline for 21 days. The frequency of individual shRNAs was quantified at baseline, after establishment of SSX2-induced senescence and in a non-senescence growth control, using next generation sequencing (NGS). **g** shRNAs enriched in doxycycline-treated samples (two replicates) compared to untreated samples and baseline were identified that might target genes essential for SSX2-induced senescence. SSX and Mediator shRNAs enriched more than fivefold in doxycycline (DOX)-treated samples, compared to untreated and baseline samples are highlighted. As expected, SSX shRNAs were identified together with shRNAs targeting genes encoding multiple subunits of the Mediator complex. Data represents the mean ± SD for three biological replicates. ***< 0.0001. Scale bars = 25 µm

Functional genetic screening using shRNA libraries is a powerful tool for identification of genes essential for specific cellular phenotypes. We applied this technique to a previously established model^10^, wherein MFC7 cells with ectopic expression of SSX2 (Fig. 1a, b) undergo a senescence response, evident by loss of proliferation, enhanced beta-galactosidase activity, and increased cell size (Fig. 1c–e). MCF7 cells were transduced with lentivirus carrying an shRNA library targeting 4922 human genes with 4–6 shRNAs per gene (Fig. 1f). Since SSX proteins are DNA-binding factors with a role in structural regulation of chromatin and might induce senescence through a chromatin-associated mechanism, we selected a library constructed by Cellecta that was focused towards genes encoding DNA binding molecules. Following lentiviral infection with shRNA-encoding plasmid constructs, the cells were treated with doxycycline to induce SSX2 expression. Subsequently, the cells were cultured for 21 days to allow senescence development (approximately 7 days) (Fig. 1f) and outgrowth of cell clones that did not undergo senescence-associated cell growth arrest due to shRNA-mediated knockdown of specific genes. Cells without SSX2 expression were cultured in parallel to control for genes that affect MCF7 cell growth in general. Massive parallel sequencing of the baseline sample (MCF7 cells infected with the library), post-senescence samples and non-senescence control samples in two replicates identified a list of shRNAs enriched in post-senescence samples that might target genes essential for the development of SSX2-induced senescence (Fig. 1g). There was a high concordance between the two replicates (Fig. 1g). Eleven genes that were targeted by at least two shRNAs enriched more than fivefold in the post-senescence samples, compared to baseline samples and the non-senescence samples, were considered potential mediators of SSX-induced senescence (Table S1). The presence of several shRNAs targeting *SSX2* and homologous SSX genes was indicative of a successful approach (Fig. 1g).

### Mediator complex subunits are essential for SSX2-induced senescence

**Fig. 2:**
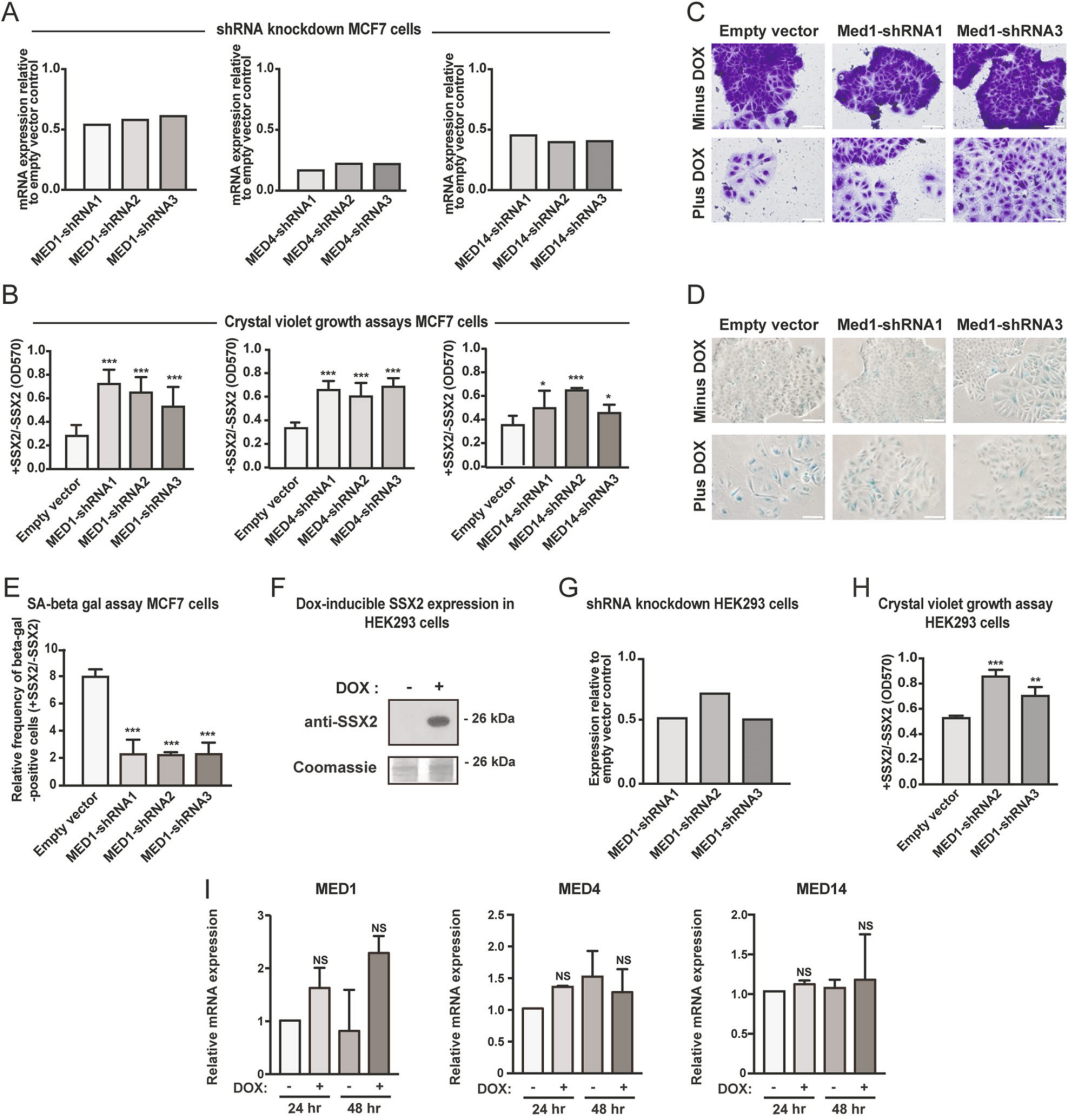
Reduced levels of Mediator subunits prevents SSX2-mediated senescence. **a** MCF7 cells with doxycycline (DOX)-inducible SSX2 expression were transduced with plasmids encoding shRNAs specific for genes encoding the Mediator subunits MED1, MED4, or MED14. Relative expression levels of *MED1*, *MED4*, and *MED14* were determined using quantitative RT-PCR. **b**–**e** MCF7 cells with *MED1*, *MED4*, or *MED14* shRNA-mediated knockdown were treated with doxycycline to induce SSX2 expression and cultured for 7 days. Cell growth was then measured using a crystal violet cell staining followed by quantification at OD570 (**b**). Representative pictures are shown (**c**). The frequencies of senescent cells were identified by beta-galactosidase assay (**d**) and quantified (**e**). **f** HEK293 cells were previously shown to acquire a senescence-like morphology and growth arrest in response to doxycycline-inducible expression of SSX2^10^. Expression of SSX2 was validated by Western blotting. Coomassie staining of the membrane was included as loading control. **g** We also knocked down *MED1* expression in this model and determined the efficiency by quantitative RT-PCR. **h** The effect of *MED1* knockdown on cell growth was evaluated 7 days after induction of SSX2 expression by crystal violet cell staining followed by quantification at OD570. **i** The expression levels of MED1, MED4, and MED14 were quantified by RT-PCR in MCF7 cells with and without doxycycline-induced SSX2 expression. Data represent the mean ± SD for three biological replicates. ns = non significant. ***< 0.0001; **< 0.001; *< 0.01. Scale bars = 100 µm. **a** and **g** show the average of three technical replicates therefore no standard deviations are shown

Among the 11 genes identified, the most prominent were subunits of the Mediator complex (Fig. 1f; Supplementary Tables 1 and 2). Mediator complex subunits 1 and 4 (MED1 and MED4) were represented by three and two shRNAs, respectively, but single shRNAs targeting *MED9*, *MED14*, *MED21*, *MED22*, *MED23*, and *MED26* were also highly enriched in post senescence samples (Table S2). This strongly suggested that the Mediator complex is important for SSX2-induced senescence. To validate this, we targeted the expression of *MED1*, *MED4*, and *MED14* by novel sets of shRNAs in a single gene-knockdown format. Despite testing many different shRNAs targeting *MED1*, *MED4*, and *MED14*, a general knockdown efficiency of only approximately 50–70% (Fig. 2a) suggested that complete knockdown of these genes was not tolerated by the cells, which is in accordance with the central role of the Mediator complex in transcriptional regulation. However, reduced levels did not seem to perturb cell growth (Fig. S1). Interestingly, this reduction in expression level was sufficient for the MCF7 cells to bypass SSX2-induced, senescence-associated growth arrest and confirmed the role of MED1, MED4, and MED14 in this phenotype (Fig. 2b). In addition, a strong reduction in the frequency of enlarged (Fig. 2c) and senescence-associated, beta-galactosidase-positive cells (Fig. 2d, e) was observed following knockdown of *MED1* expression. To investigate whether the role of the Mediator complex in SSX2-induced senescence was cell type-specific, we knocked down *MED1* with shRNAs in the HEK293 cell line model with inducible SSX2 expression (Fig. 2f)^10^. HEK293 cells have been demonstrated to be prone to undergo senescence^33^. As for MCF7 cells, the level of *MED1* expression was reduced to about 50% (Fig. 2g) and reduced levels of *MED1* facilitated bypassing SSX-induced senescence-associated growth arrest (Fig. 2h), suggesting a more general role of the Mediator complex in SSX2-induced senescence. The expression levels of individual Mediator Complex subunits were not changed during SSX2-induced senescence (Fig. 2i). We also tested the ability of SSX2 to induce senescence in IMR90 fibroblast cells, which can undergo other types of senescence. SSX2 reduced the growth of the cells, but did not induce senescence characteristics such as enlarged cell size and beta-galactosidase activity (Fig. S2).

### MED1 is important for SSX2-mediated activation of p21 transcription

**Fig. 3:**
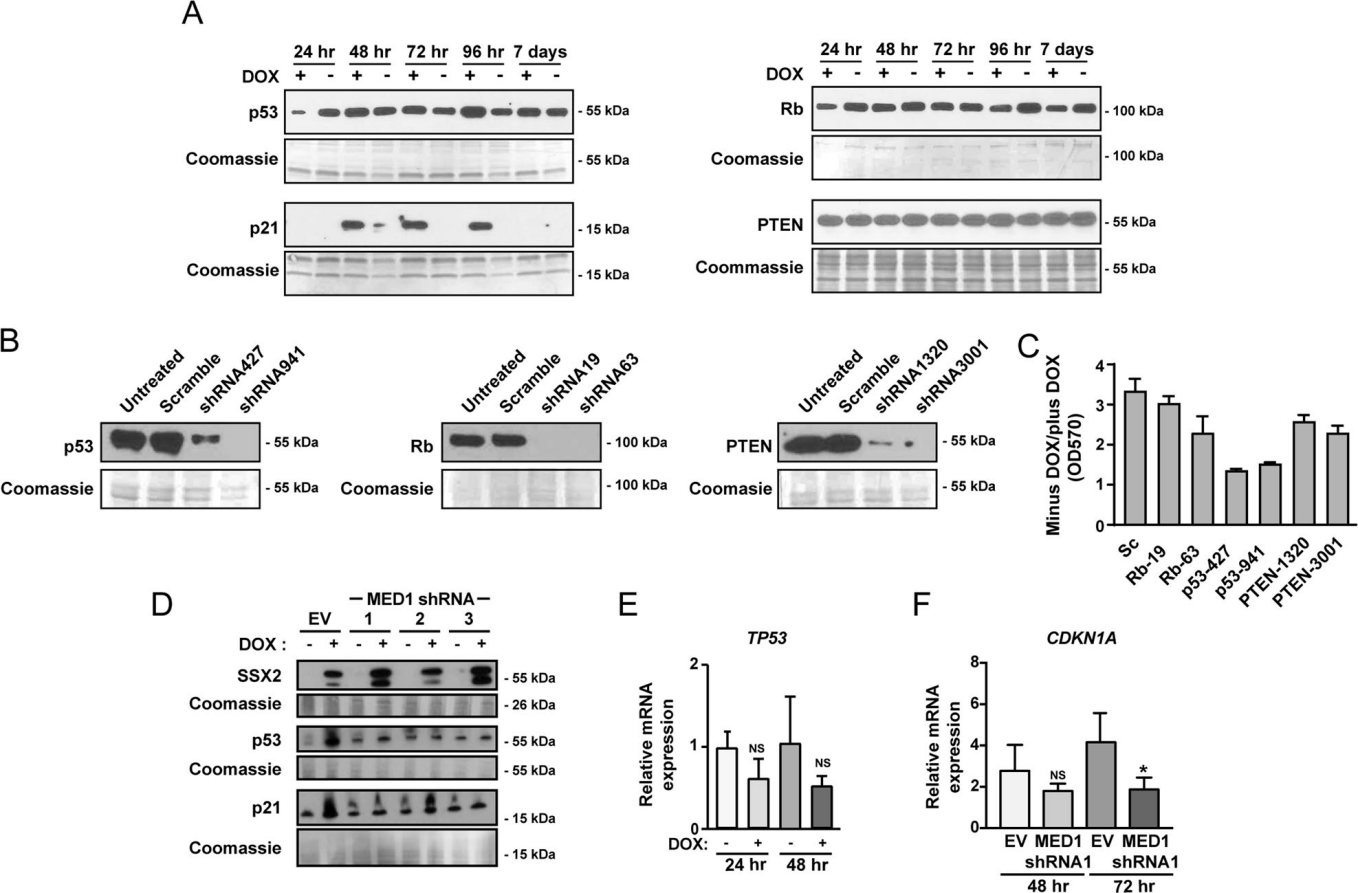
The effect of MED1 in SSX2-mediated activation of p53–p21. **a** The SSX2-mediated induction of important tumor suppressors was investigated using Western blotting at indicated time points after addition of doxycycline to induce SSX2 expression. Coomassie staining of the membrane was included as loading control. **b** The expression of genes encoding p53, Rb, and PTEN was knocked down using lentiviral transductions with shRNA expression plasmids and confirmed with Western blotting. Coomassie staining of the membrane was included as loading control. **c** The effect of p53, Rb, and PTEN knockdown on SSX2-mediated growth arrest was investigated using crystal violet staining aften 6 days of doxycycline-induced SSX2 expression and quantified at OD570. **d** The effect of 6 days of doxycycline-induced SSX2 expression on the levels of p53 and p21 in MCF7 cells with and without reduced *MED1* expression was investigated using Western blotting. Coomassie staining of the membrane was included as loading control. **e**, **f** The expression levels of TP53 (**e**) and CDKN1A (**f**) were quantified by RT-PCR in MCF7 cells with and without doxycycline-induced SSX2 expression. *< 0.01

In a previous study, we showed that SSX2-induced senescence was associated with increased levels of p53 and its downstream target, the cycline-dependent kinase inhibitor p21^10^. There is strong evidence that p53 activity and the p53–p21 axis are major regulators of senescence in human cells^34^. Thus we investigated the role of p53 and two other important tumor suppressor proteins, Rb and PTEN, in SSX2-mediated senescence in MFC7 cells (Fig. 3). SSX2 expression was associated with increased levels of p53 and its downstream target p21 (Fig. 3a). Rb was slightly reduced a later time points (4 and 7 days), while PTEN remained unchanged (Fig. 3a). Knockdown of *TP53*, *RB1*, and *PTEN* confirmed the importance of p53 in SSX2-mediated senescence (Fig. 3b, c). Thus we investigated whether bypass of the SSX2-induced senescence response by knockdown of *MED1* expression would reduce levels of p53 and p21. Indeed, we found that MCF7 cells with shRNA-mediated knockdown of *MED1* levels during induction of SSX2 expression exhibited reduced levels of both p53 and p21 (Fig. 3d). Interestingly, SSX2 expression did not enhance mRNA levels of *TP53* (encoding p53), suggesting that increased protein levels were achieved through protein stabilization (Fig. 3e). The role of MED1 in SSX2-induced activation of the p53–p21 axis was further indicated by reduced *CDKN1A* (encoding p21) expression in MCF7 cells with *SSX2* expression and *MED1* knockdown (Fig. 3f).

### Mediator complex subunits are not essential for the senescence response in general

**Fig. 4:**
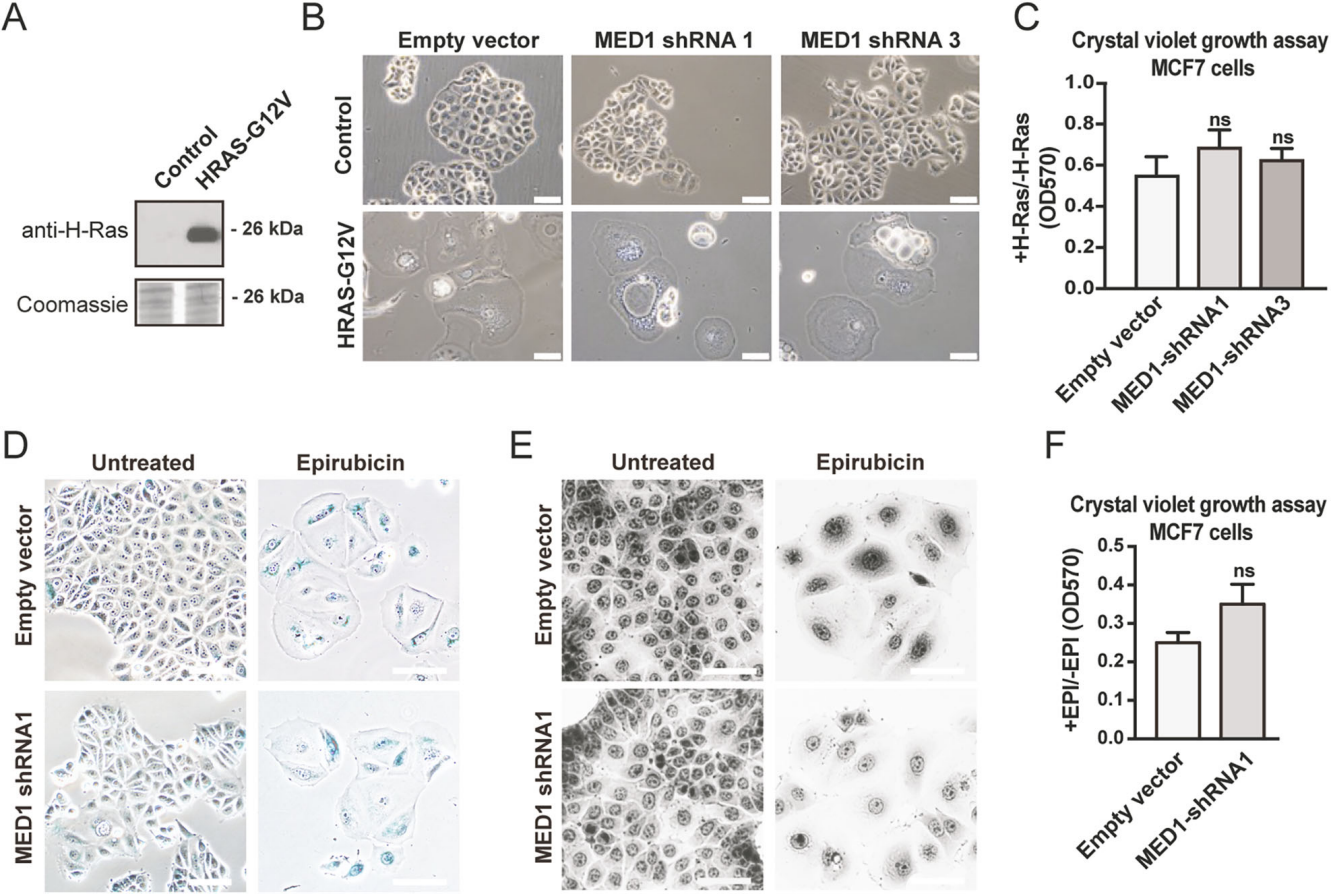
MED1 is not important for H-Ras oncogene- and epirubicin-induced senescence. **a** MCF7 cells stably transduced with *MED1* shRNA expression vectors or empty vector control were transduced with an H-Ras G12V expression plasmid or empty control. H-Ras G12V expression was confirmed by Western blotting. Coomassie staining of the membrane was included as loading control. **b**, **c** In 3–4 days, the cells expressing H-Ras G12V acquired a senescent phenotype (**b**) and underwent growth arrest (**c**). **d**–**f** Reduced levels of MED1 does not prevent enhanced beta-galactosidase activity (**d**), the acquisition of senescent morphology (**e**), or senescence-associated growth arrest (**f**) in epirubicin treated cells. Cells were analyzed 7 days after treatment. Data represent the mean ± SD for three biological replicates. ns = non significant. Scale bars = 100 µm

Having demonstrated a role for the Mediator complex in SSX2-mediated senescence in MCF7 and HEK293 cells, we investigated its role in SSX2-independent senescence using a model of H-Ras oncogene-induced senescence in MCF7 cells^35^. Following overexpression of H-Ras G12V in MCF7 cells (Fig. 4a), a senescent phenotype and growth arrest similar to *SSX2*-expressing MCF7 cells was observed (Fig. 4b). Next, we investigated whether knockdown of *MED1* expression in this model diminished senescence development in response to H-Ras expression. Interestingly, no significant effect of *MED1* knockdown on senescence-associated growth inhibition or morphological changes induced by H-Ras was observed (Fig. 4a, b), suggesting that the Mediator complex is important for the cellular response to SSX2, but not to H-Ras.

We also investigated the role of Mediator in a drug-induced senescence model, where MCF7 cells undergo senescence in response to brief Epirubicin treatment (Fig. 4d–f). Knockdown of MED1 in this model did not affect the Epirubicin-induced increase in beta-galactosidase activity and increase in cell size (Fig. 4d, e). Nor did it significantly reduce senescence-associated loss of proliferation, although there was a tendency towards such an effect (Fig. 4f).

These results suggest a more specific role for the Mediator complex in the molecular function exerted by SSX2 in MCF7 cells, which ultimately leads to senescence. It could also be due to mechanistic differences in the senescence response to SSX2 and H-Ras/Epirubicin, where only the former may depend on the conserved activity of the Mediator complex.

### Expression of Mediator subunits is deregulated in melanoma

**Fig. 5:**
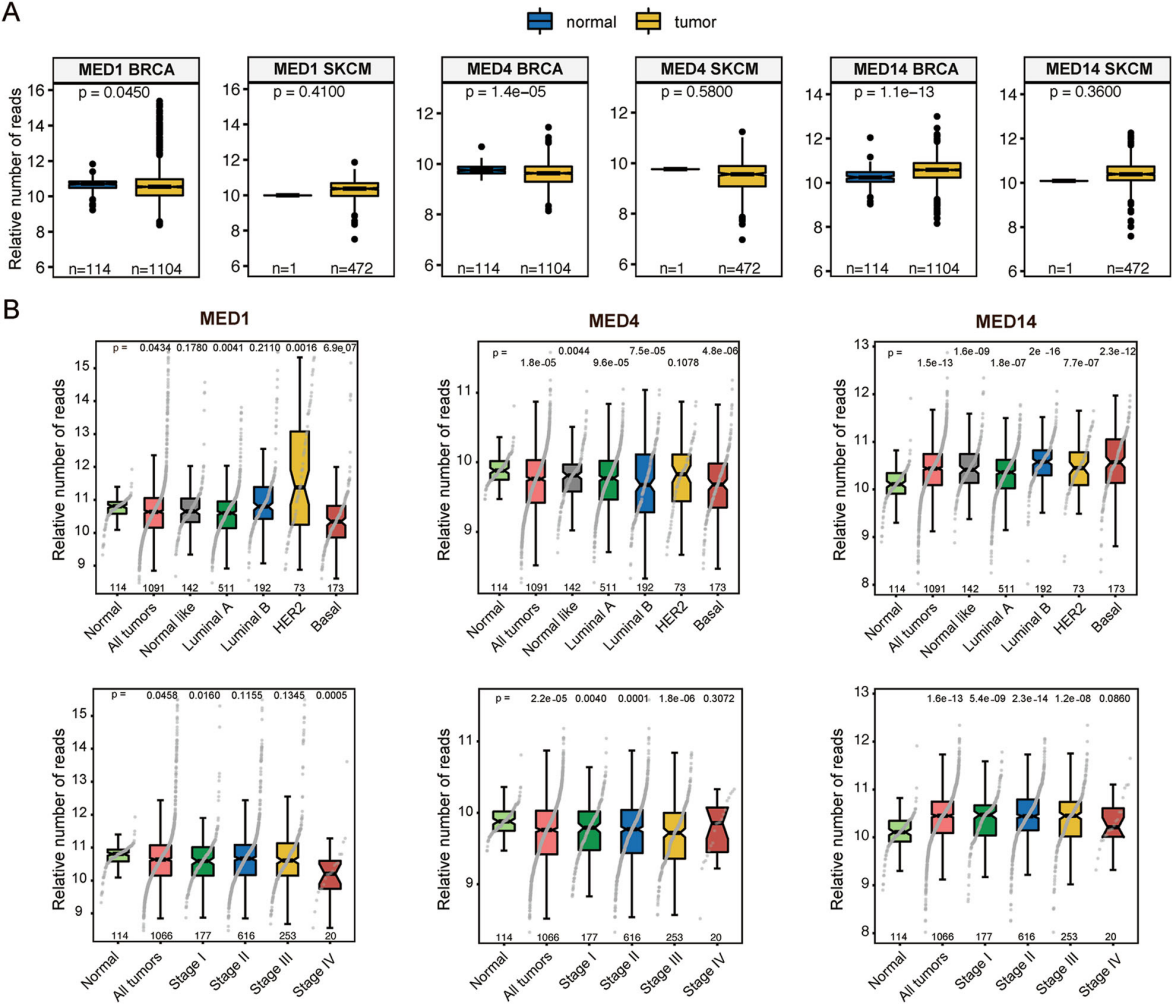
Expression of Mediator subunits in melanoma and breast cancer tumors. The expression levels of genes encoding the Mediator subunits MED1, MED4, and MED14 were examined in breast cancer and melanoma using RNA-seq data from the TCGA repository. Results are presented as relative expression levels (normalized number of reads). **a** Expression of *MED1*, *MED4*, and *MED14* in normal breast epithelia and breast tumors (BRCA) and in normal skin and melanoma tumors (SKCM). **b** Expression of *MED1*, *MED4*, and *MED14* indifferent subtypes and stages of breast cancer. Number of specimens are indicated. The *p* values were calculated using the (unpaired) Mann–Whitney *U* test

Our results showing that reduced expression of *MED1*, *MED4*, and *MED14* prevented SSX2-mediated senescence suggests that diminished Mediator complex activity might be a compensating factor for maintaining proliferation in response to SSX expression in tumors. To this end, we investigated the potential deregulation of the Mediator complex subunits in clinical tumor samples. A direct correlation between the Mediator complex activity and SSX expression is complicated by the many different Mediator complex subunits and SSX members, and thus decreased activity of the Mediator complex in a particular tumor could be facilitated by reduced expression of any of the subunits. In agreement, there was no obvious anti-correlation between expression of SSX2 and different Mediator subunits in tumors (Fig. S3). Thus, instead of a direct correlation of SSX2 and Mediator complex levels, we investigated the potential deregulation of Mediator complex subunits in various cancer types, including breast cancer and melanoma, which often express SSX2 and other SSX proteins (Fig. 5 and S4). In general, TCGA RNA-seq data showed that expression of *MED1*, *MED4*, and *MED14* are not lost in tumors, but varies considerably between tumor specimens compared normal (Fig. 5). In some cancer types, the expression was reduced in tumors compared to normal tissues, but in others it was increased. In breast cancer, *MED1* and *MED4* expression was significantly reduced in tumors, while *MED14* was increased (Fig. 5a). In melanoma, *MED1* and *MED14* was increased, while *MED4* was reduced (Fig. 5a). Strikingly, *MED1* expresssion was significantly enhanced in HER2-positive breast cancers, while reduced in the basal-like subtype (Fig. 5b). *MED1* and *MED4* expression was generally slightly reduced with breast cancer progression (Fig. 5b), while *MED14* expression was generally increased (Fig. 5b).

**Fig. 6:**
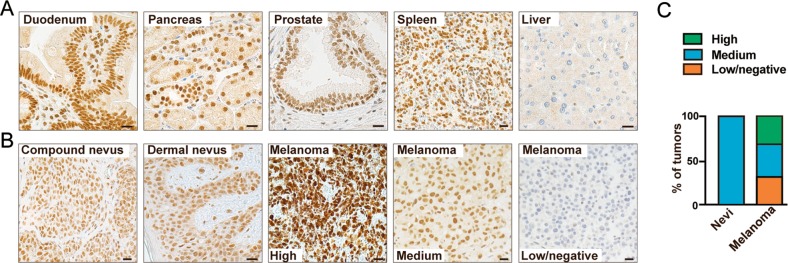
The level of MED1 changes during melanoma tumorigenesis. **a** The level of MED1 was examined using immunohistochemical staining in normal tissues. Medium levels of expression were observed in all tissues except the liver. Representative stainings are shown. **b** The level of MED1 was also investigated in 39 dermal and compound nevi and in 74 primary and metastatic melanoma tumors. The level of MED1 was quantified as high, medium, or low/negative. Representative pictures are shown. **c** Quantification of the results from *A* and *B* show consistent MED1 expression levels among nevi and diverging levels among tumors. Scale bars: *A* = 100 µM, *B* = 25 µm

We also investigated the expression of MED1 using immunohistochemical staining. First, we validated the specificity of the MED1 antibody by staining a panel of normal tissues. As expected, that MED1 was detected in all tissues except the liver, and was localized predominantly in the nuclei (Fig. 6a), in accordance with data from the Human Protein Atlas (www.proteinatlas.org). Next, we investigated if MED1 expression is deregulated in association with loss of the senescence response during tumorigenesis. This was most appropriately done in melanoma, where premalignant specimens are easily accessible (i.e., nevi). Thus, we quantified MED1 levels in a panel of benign nevi (*n* = 39) and melanoma tumors (*n* = 74) using immunohistochemical staining. Melan-A was stained on parallel tissue sections for identification of melanocytic lesions (for an example see Fig. S5). The analysis demonstrated consistent medium levels of MED1 expression in the nuclei of all melanocytic cells of benign dermal and compound nevi, whereas expression in the nuclei of melanomas was much more inconsistent, including specimens with high, medium, or low/no expression (Fig. 6b). Within both benign and malignant tumors, MED1 exhibited a relatively homogenous expression pattern (Fig. 6b). Immunohistochemical staining of MED1 showed that 31% and 27% of melanoma specimens exhibited high or low/no expression, respectively (Fig. 6c). These results were consistent with a previous study reporting that MED1 was often either upregulated or downregulated in melanoma^36^, which also showed that low MED1 expression correlated with a highly tumorigenic phenotype. Multiple other studies have also demonstrated that MED1^31,37–41^ and Mediator subunits are deregulated in melanoma and other cancer types^31,42–46^. The observed differences in expression of different MEDs may translate into diffences in the activity of the Mediator complex to support tumorigenesis and specific phenotypes, such as SSX2 expression.

## Discussion

The SSX2 protein, like well-established oncogenes such as B-Raf, Ras, Cyclin D, and Myc, supports cancer cell proliferation, but at the same time induces senescence-associated growth arrest when ectopically expressed^10^. This paradox most likely reflects the need for additional alterations that prevent or bypass the senescence response. Identifying such alterations is an important step toward a better understanding of tumor initiation. In this study, we used a functional genetic screening approach to identify genes that, when reduced in expression, prevent a SSX2-induced senescence response. Interestingly, we identified several subunits of the Mediator complex as essential for this phenotype to develop. Notably, it was only possible to achieve 50–80% knockdown of *MED1*, *MED4*, and *MED14*, and cells with reduced expression exhibited reduced growth. This suggested that complete loss of Mediator was not tolerated by the cells, which is in accordance with other studies showing that complete knockout of Mediator subunits is embryonically lethal and impairs cell growth and cell cycle progression^3^, but in disagreement with another study showing that complete *MED1* knockdown is indeed possible^47^. However, a reduction in the levels of Mediator subunits was enough to prevent SSX2-induced senescence in MCF7 and HEK293 cells. Interestingly, SSX2 did not induced a senescence response in IMR90 fibroblast cells, suggesting that that the ability of SSX2 to induce senescence is dependent on cell types or specific molecular patterns.

The Mediator complex has a multifaceted role in integrating cellular signals into changes into transcriptional reprogramming, but has not been described in association with senescence. Senescence is a complex phenotype, which, in addition to loss of proliferative signaling, also involves major changes to the chromatin regulation, metabolism and structure of the cell^28^. A role for Mediator in senescence development was, therefore, not unexpected. Moreover, the Mediator complex has previously been linked to the function of p53. In one study, Mediator subunits, including cofactor CDK8, were shown to be recruited to the *CDKN1A* locus together with p53 and modulate the strength of p53-mediated transcriptional activation^48^. In another study, p53 was demonstrated to activate transcription by directly binding and changing the conformation of the Mediator complex^49^. Thus, there seems to be an intimate relationship between p53-activity and the functions of the Mediator complex. Whether interactions with the Mediator complex also stabilizes p53, as indicated here (Fig. 3a, b), remains to be determined. The prominent role of p53 in the senescence response and the direct link between the Mediator complex and p53 activity further suggested a role for the Mediator complex in senescence. However, knockdown of *MED1* in other models of senescence in which MCF7 cells undergo senescence in response to the H-Ras oncogene or Epirubicin suggested that the activity of the Mediator complex per se was not instrumental for development of senescence (Fig. 4). Our data linked the Mediator complex to the SSX-mediated senescence.

There are several posibile ways that the Mediator complex may mediate SSX2-induced senescence. The Mediator complex may be central for the senescence response to SSX2 perhaps by regulating the gene expression of central factors. This does not seem to include regulation of *TP53* as this gene was not significantly induced in response to SSX2 expression. The Mediator complex may also be important for SSX2 functions, which include but may not be limited to, chromatin regulation/rearrangement. The latter is supported by our recent data demonstrating that SSX proteins bind and rearrange chromatin in cancer cells, and it is likely that the effect of the SSX2 protein on chromatin structure and gene expression is what initiates SSX2-mediated senescence. Importantly, SSX proteins seem to play an antagonistic role in Polycomb group (PcG) gene repression, suggesting that ectopic expression of SSX molecules may release PcG-repressed genes^17^ and facilitate binding of RNA polymerase II preinitiation complex (PIC) to change the transcription landscape. In agreement with this, PcG complexes have been demonstrated to directly inhibit the assembly of the PIC^50^. Thus, the Mediator complex may facilitate the SSX-mediated changes in gene expression that initiates a senescence response. Another possibility is that the SSX2-mediated structural rearrangement of chromatin involves the Mediator complex, which is important for several aspects of chromatin organization.

An important implication of our results is that reduced levels of MED1, MED4, or MED14 may support specific cancer-associated alterations (e.g., SSX2 expression). This observation is interesting in the light that the expression of multiple subunits of the Mediator complex, including MED1, MED4, or MED14, is frequently dysregulated in different types of cancer.

## Materials and methods

### Cells and culture conditions

MCF7-SSX2, HEK293-SSX2 cells were grown in DMEM (Sigma Aldrich, Brondby, Denmark), 10% fetal bovine serum (FBS) and penicillin/streptomycin, supplemented with 6 ng/ml insulin (MCF7). When relevant, cell identities according to ATCC were verified using DNA fingerprinting by short tandem repeat analysis (Cell IDTM system, Promega) and tested for mycoplasma (MycoAlert, Mycoplasma detection kit, Lonza). MCF7 cells were were treated for two hours with 1 µM Epirubicin to induce senescence. IMR90 cells were cultured in EMEM (Sigma Aldrich, Brondby, Denmark), 10% FBS and penicillin/streptomycin. IMR90 cells were transduced with a lentiviral vector for expression of doxycycline-inducible SSX2 expression as previously described^14^.

### shRNA library preparation

The shRNA screen was conducted using the Decipher module 3 pooled shRNA library (Cellecta Inc.), which contains 27500 shRNAs targeting 4922 human genes (i.e., 5–6 shRNAs per gene). The library was prepared as lentiviral particles by co-transfection of HEK293T cells with second-generation packaging plasmids psPAX2 and pMD2.G (Cellecta Inc.) using Lentifectin transfection reagent (Biocat, Germany). Lentiviral particles were harvested after 72 h and concentrated using Lenti-X concentrator (Takara Clontech).

### Functional genetic screening with a pooled shRNA library

MCF7 cells with doxycycline-inducible expression of SSX2 (i.e., MCF7-SSX2) were infected with lentiviral particles carrying the pooled shRNA library in the presence of 5 µg/ml polybrene to obtain 40% transduced cells. Cell numbers were adjusted to maintain a library representation of >400. Cells were seeded in culture plates and selected with 0.2 µg/ml puromycin for 5 days. Cells were harvested and reseeded at a density of approximately 2.2 × 10^3^ cells/cm^2^ in four replicates. The remaining cells were frozen for later use as baseline samples. Two replicates were supplemented with 100 ng/ml doxycycline to induce SSX2 expression and cells were cultured for 21 days with addition of fresh media and doxycycline every three days. Next, gDNA was isolated from the baseline sample, no-doxycycline controls and doxycycline-treated cells using the Blood and cell culture maxi kit (Qiagen) according to the manufacturers recommendations and sheared into smaller fragments using a tip-sonicator set at 20% for 2 × 10 s. shRNA library barcodes were amplified with nested polymerase chain reaction (PCR) (see Table S3 for primers) using Titanium Taq DNA polymerase (Clontech-Takara). For the first PCR, 200 µg of gDNA were used to maintain library coverage and reactions were run for 16 cycles. A fraction of the product (1/80) was reamplified with indexing primers for 14 cycles in a second PCR. Products were purified from a 3% agarose gel using the Nucleospin gel and PCR cleanup kit (Macherey–Nagel, Dueren, DE) and quantified with Quant-iT picogreen reagent (firma). Samples were pooled and subjected to end sequencing using a Hiseq sequencer (Illumina, San Diego, CA, USA). Sequencing data were aligned to the library of barcode identifiers using the Barcode deconvoluter software (Cellecta) and normalized to total reads. Only barcodes identified with more than 100 reads in the doxycycline-treated samples were considered in the further analysis comparing barcode frequencies from the baseline sample, no-doxycycline controls and doxycycline-treated cells.

### Growth assays

Cells were seeded in 6-well plates at a density of 5000 cells/well, and after 24 h 100 ng/ml of doxycycline was added. After approximately 10 days, plates were washed in PBS and stained with 5 mg/ml crystal violet in 25% methanol, H_2_O for 15 min. After 3 washes in H_2_O, plates were air-dried and photographed or the crystal violet stain was solubilized in buffer consisting of 29.41 mg/ml citrate in 50% ethanol, H_2_O and quantified on a Spectramax Paradigm reader (Molecular Devices) at OD570.

### Senescence-associated beta-galactosidase assay

Senescence-associated beta-galactosidase activity was measured at pH 6.0 using a senescence beta-galactosidase staining kit (Cell Signaling) according to the manufacturer’s recommendations.

### Western blotting

Extracts were made from monolayers of cells with RIPA buffer, resolved by 4–20% sodium dodecyl sulfate polyacrylamide gel electrophoresis and electroblotted onto a polyvinylidene fluoride membrane. The membrane was blocked in PBS, 0.1% Tween-20, and 5% non-fat dry milk powder and then incubated with anti-SSX2-4 (1:3000)^51^, p53 (Santa Cruz Biotechnology, sc-126, 1:500), p21 (Santa Cruz Biotechnology, sc-53870, 1:1000), Rb (Cell Signaling, #9309, 1:2000), PTEN (Cell Signaling, #9188, 1:1000), or anti-H-Ras (Santa Cruz Biotechnology, sc-520, 1:1000). The blot was further stained with horseradish peroxidase-conjugated goat anti-mouse IgG (DakoCytomation Denmark A/S, Glostrup, Denmark) and developed with an ECL Western Blot kit (Amersham Biosciences, Hilleroed, Denmark, 1:100.000). All antibody incubation and washing steps were carried out in PBS, 0.1% Tween-20.

### Quantitative RT-PCR

RNA was purified from cells using RiboZol (VWR) followed by cDNA synthesis using the RevertAid Premium Reverse Transcriptase kit from Fermentas. Quantitative real-time PCR was performed using SYBR green based expression analysis (Applied Biosystems) in combination with Quantitect primers: MED1 #QT00076356, MED4 #QT00039158, MED14 #QT00063490, PUM1 #QT00029421, CDKN1A (#QT00062090), and TP53 (#QT00060235).

### shRNA knockdown

Annealed oligoes (Table S4) were ligated into the pSico plasmid using standards methods. The shRNA plasmids were prepared as lentivirus by cotransfection with pMD2.G, pRSV-Rev, pMDL g/p RRE (kindly provided by the Trone Lab through Addgene, Cambridge, UK) into HEK293T cells using Lentifectin (Abmgood, Richmond, Canada). Virus was harvested from the supernatant after 2 and 3 days, filtered, precipitated with PEG and resuspended in PBS. For *TP53*, *CDKN1A* and *PTEN* knockdown, pLKO1 shRNA plasmids were purchased from Sigma Aldrich and prepared as lentivirus as described above.

### Indirect immunofluorescence

Cells grown on coverslips were fixed in 4% formaldehyde, permeabilized in 0.2% Triton X100, PBS and blocked in 3% bovine serum albumin (BSA), PBS. Immunostaining was done with anti-SSX2/SSX3 (clone 1A4; Sigma Aldrich; 1:100) in 1% BSA, PBS, and goat anti-mouse IgG (H+L) cross-adsorbed Alexa Fluor 488/568 (Thermo Fisher Scientific). Cells were mounted under cover slides with ProLong Gold Antifade with DAPI (Life Technologies) and imaging was performed with an Olympus IX73 microscope fitted with a PlanApo N 60X/1.42 oil objective.

### Immunohistochemical staining

Tissues sections were deparaffinized and treated with 1.5% H_2_O_2_ in Tris-buffered saline (pH 7.5) for 10 min to block endogenous peroxidase activity. Tissues were then washed in TNT buffer (0.1 m Tris, 0.15 m NaCl, 0.05% Tween-20, pH 7.5) and subjected to antigen retrieval with different protocols, including microwave boiling for 15 minutes in (1) T-EG buffer (10 mm Tris, 0.5 mm EGTA, pH 9.0), (2) 10 mm citrate buffer (pH 6.0), or (3) Dako Target retrieval solution (Dako S1699), or proteolytic treatment using (4) 0.05% protease type XIV (pronase E, Sigma, cat. no. P5147) in TBS (pH 7.0) for 15 minutes at 37 °C or (5) 0.4% pepsin (Sigma, Cat. No.: P7012) in 0.01 m HCl for 20 min at 37 °C. Optimal atigen retrieval was achieved with microwave boiling in T-EG buffer for 15 min. Sections were then incubated with rabbit anti-TRAP220/MED1 antibody (NB100-2574; Novus Biologicals) or mouse anti-Melan A (Clone A103; Ventana Medical Systems) diluted in antibody diluent (S2022, DAKO Cytomation, Glostrup, Denmark) for 1 h at room temperature. Subsequently, sections were washed with TNT, incubated with EnVision Flex/HRP+ for 30 minutes, washed again and incubated with 3,3′-diaminobenzidine (DAB)+ substrate-chromogen for 10 min. Following another wash with H_2_O, sections were counterstaining with Mayers hematoxylin before mounting in AquaTex (Merck Inc., Whitehouse Station, NJ, USA). Normal tissues analyzed for MED1 expression included: Tonsil, skin, esophagus, parotis, lung, thyroid, spleen, liver, gall bladder, colon, duodenum, muscle, testis, prostate, bladder, kidney, uterus, and placenta.

### Statistical testing

Statistical testing was done using the *t* test to compared two groups or the one-way ANOVA for comparing multiples groups (Figs. 1–4). For statistical testing of larger data sets (Fig. 5), the Mann–Whitney *U* test was used.

## Supplementary information

DECLARATION OF CONTRIBUTIONS TO ARTICLE

Figure S1

Figure S2

Figure S3

Figure S4

Figure S5

Table S1

Table S2

Table S3
