# Supplementary material for: A functional genetic screen identifies the Mediator complex as essential for SSX2-induced senescence
Source: Cell Death Dis. 2019 Nov 6;10(11):841. doi: 10.1038/s41419-019-2068-1 (PMC6834653; doi:10.1038/s41419-019-2068-1)

Figure S1. The effect of MED1 shRNA knockdown on cell growth. Cells transduced with MED1 shRNAs or empty vector was grown for 7 days and cell numbers were quantified using crystal violet staining and measurement of solubilized crystals at OD570. Data represents the mean ± SD for three biological replicates.

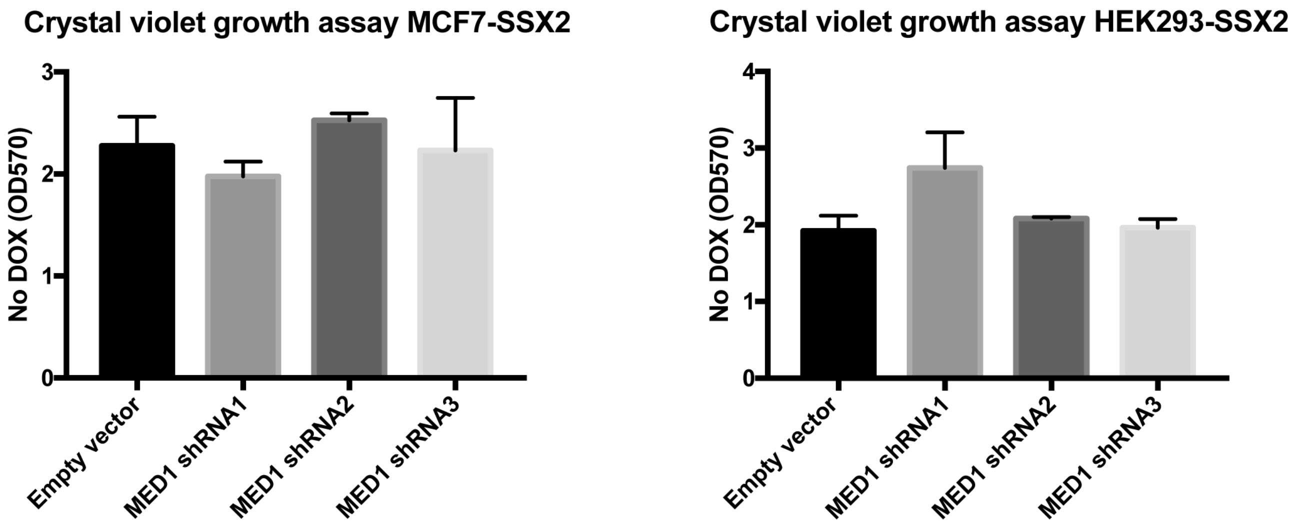

Supplement: Supplementary file 1 — DECLARATION OF CONTRIBUTIONS TO ARTICLE [file 41419_2019_2068_MOESM1_ESM.pdf]
