# Supplementary material for: A functional genetic screen identifies the Mediator complex as essential for SSX2-induced senescence
Source: Cell Death Dis. 2019 Nov 6;10(11):841. doi: 10.1038/s41419-019-2068-1 (PMC6834653; doi:10.1038/s41419-019-2068-1)

Figure S2. The effect of ectopic SSX2 expression in IMR90 fibroblast cells. IMR90 cells were transduced with lentivirus carrying a pLVX-TET-One-Puro-SSX2 expression plasmid for doxycycline (DOX)-inducible expression of SSX2. Cells were selected for 4 days with puromycin and seeded with and without 100 ng/ml doxycycline. (**A**) Expression of SSX2 in IMR90 cells was confirmed with immunofluorescent staining, which showed that 100% of cells were SSX2-positive. (**B-C**) IMR90 cells were cultured for 7 days with or without doxycycline. The frequency of senescent cells was quantified with beta-galactosidase staining (B) (percentage of positive cells are shown) and cell growth was quantified using crystal violet staining and OD570 measurement of solubilized crystals (C). Data represent the mean ± SD for three biological replicates. ** < 0.001. Scale bars = 10 µm.

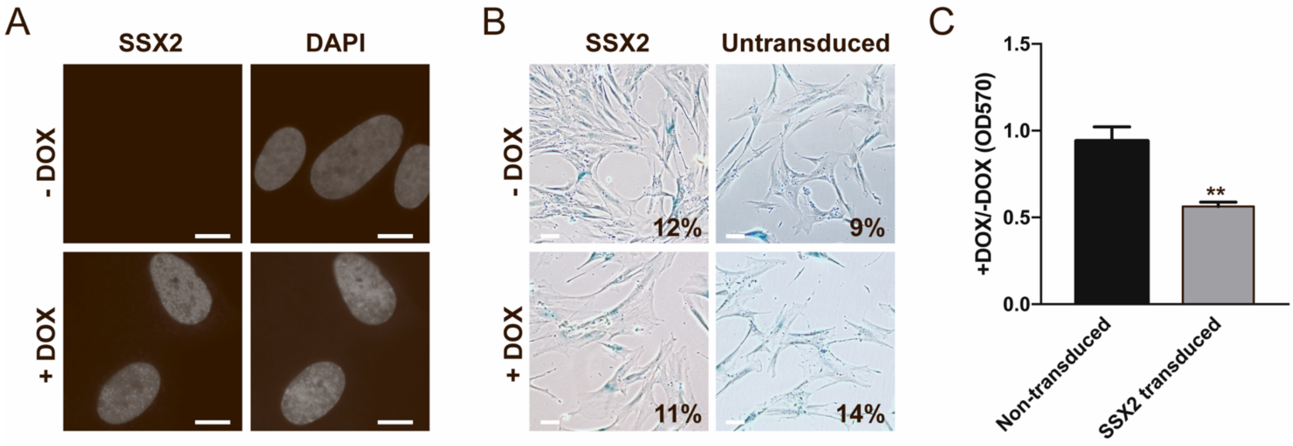

Supplement: Supplementary file 2 — Figure S1 [file 41419_2019_2068_MOESM2_ESM.pdf]
