# Supplementary material for: A functional genetic screen identifies the Mediator complex as essential for SSX2-induced senescence
Source: Cell Death Dis. 2019 Nov 6;10(11):841. doi: 10.1038/s41419-019-2068-1 (PMC6834653; doi:10.1038/s41419-019-2068-1)

**Figure S3. Analysis of the potential correlation between SSX2 and Mediator subunit expression in primary tumors of melanoma and breast cancer.** Numbers are relative reads. Data was extracted from the TCGA repository.


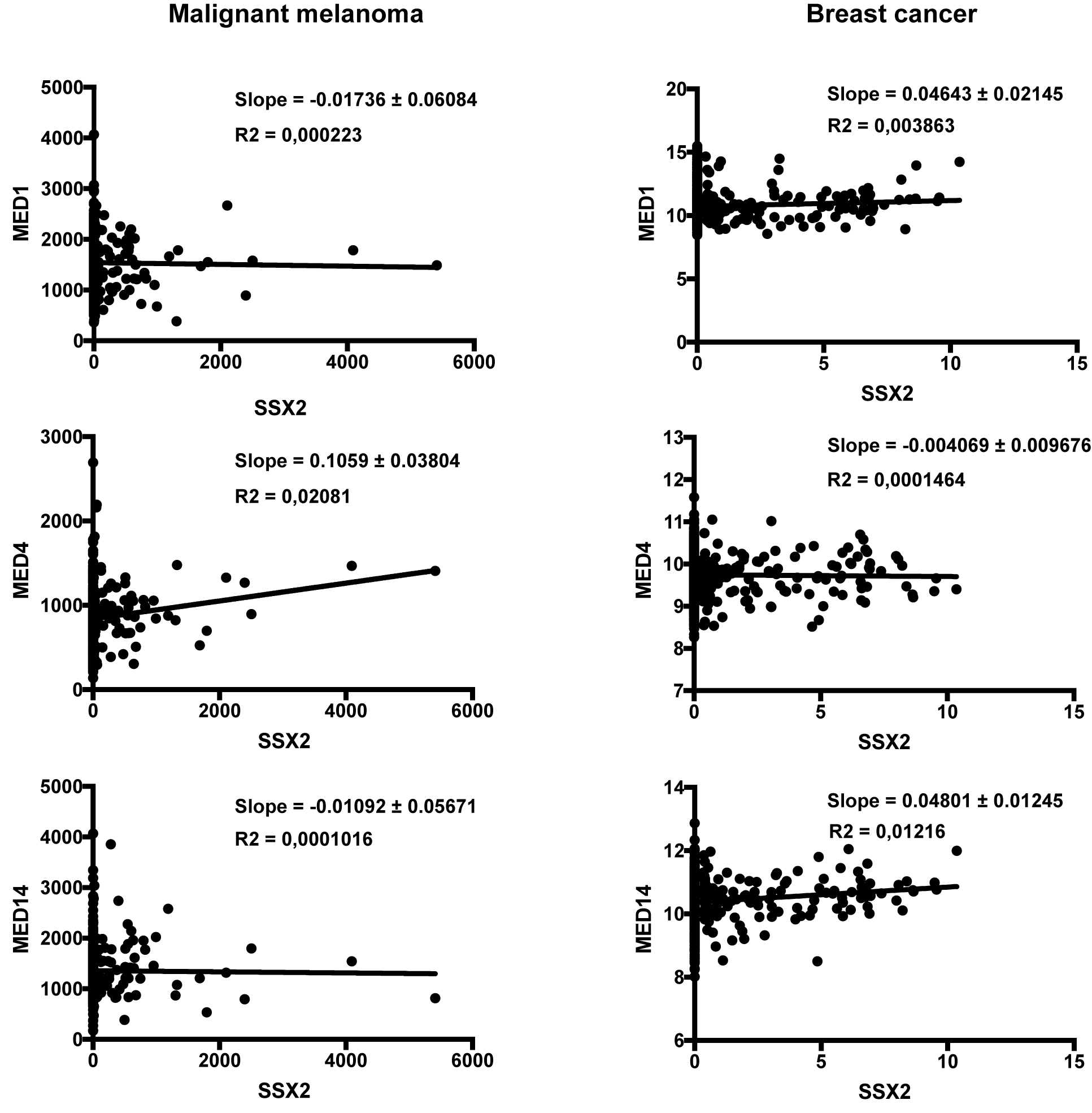

Supplement: Supplementary file 3 — Figure S2 [file 41419_2019_2068_MOESM3_ESM.docx]
