# Supplementary material for: A functional genetic screen identifies the Mediator complex as essential for SSX2-induced senescence
Source: Cell Death Dis. 2019 Nov 6;10(11):841. doi: 10.1038/s41419-019-2068-1 (PMC6834653; doi:10.1038/s41419-019-2068-1)
Supplement: Supplementary file 4 — Figure S3 [file 41419_2019_2068_MOESM4_ESM.docx]

**Figure S4. Expression of Mediator subunits MED1, MED4 and MED14 in different types of human cancer.** Data was extracted from the TCGA repository. Number of specimens are indicated. The p-values were calculated using the (unpaired) Mann-Whitney U test. ACC, Adrenocortical Cancer; BLCA, Bladder Cancer; BRCA, Breast Cancer; CESC, Cervical Cancer; CHOL, Cholangiocarcinoma (bile duct cancer); COAD, Colon Cancer; DLBC, Large B-cell Lymphoma; ESCA, Esophageal Cancer; GBM, Glioblastoma; HNSC, Head and Neck Cancer; KICH, Kidney Chromophobe; KIRC, Kidney Clear Cell Carcinoma; KIRP, Kidney Papillary Cell Carcinoma; LGG, Lower Grade Glioma; LIHC, Liver Cancer; LUAD, Lung Adenocarcinoma; LUSC, Lung Squamous Cell Carcinoma; MESO, Mesothelioma; OV, Ovarian Cancer; PAAD, Pancreatic Cancer; PCPG, Pheochromocytoma & Paraganglioma; PRAD, Prostate Cancer; READ, Rectal Cancer; SARC, Sarcoma; SKCM, Melanoma; STAD, Stomach Cancer; TGCT, Testicular Cancer; THCA, Thyroid Cancer; THYM, Thymoma; UCEC, Endometrioid Cancer; UCS, Uterine Carcinosarcoma; UVM, Ocular melanoma.
