# Supplementary material for: A functional genetic screen identifies the Mediator complex as essential for SSX2-induced senescence
Source: Cell Death Dis. 2019 Nov 6;10(11):841. doi: 10.1038/s41419-019-2068-1 (PMC6834653; doi:10.1038/s41419-019-2068-1)
Supplement: Supplementary file 5 — Figure S4 [file 41419_2019_2068_MOESM5_ESM.docx]

**Figure S5.** **Representative pictures of Melan A and MED1 immunohistochemical staining of parallel sections of nevi.** Scale bars = 100 µM
